# Gene × Environment Interactions in Schizophrenia: Evidence from Genetic Mouse Models

**DOI:** 10.1155/2016/2173748

**Published:** 2016-09-20

**Authors:** Paula Moran, Jennifer Stokes, Julia Marr, Gavin Bock, Lieve Desbonnet, John Waddington, Colm O'Tuathaigh

**Affiliations:** ^1^School of Psychology, University of Nottingham, Nottingham NG7 2RD, UK; ^2^School of Medicine, University College Cork, Brookfield Health Sciences Complex, College Road, Cork, Ireland; ^3^School of Life Sciences, University of Glasgow, Room 220, Bower Building, Glasgow G12 8QQ, UK; ^4^Molecular and Cellular Therapeutics, Royal College of Surgeons in Ireland, Dublin 2, Ireland; ^5^Jiangsu Key Laboratory of Translational Research & Therapy for Neuropsychiatric Disorders and Department of Pharmacology, College of Pharmaceutical Sciences, Soochow University, Suzhou 215123, China

## Abstract

The study of gene × environment, as well as epistatic interactions in schizophrenia, has provided important insight into the complex etiopathologic basis of schizophrenia. It has also increased our understanding of the role of susceptibility genes in the disorder and is an important consideration as we seek to translate genetic advances into novel antipsychotic treatment targets. This review summarises data arising from research involving the modelling of gene × environment interactions in schizophrenia using preclinical genetic models. Evidence for synergistic effects on the expression of schizophrenia-relevant endophenotypes will be discussed. It is proposed that valid and multifactorial preclinical models are important tools for identifying critical areas, as well as underlying mechanisms, of convergence of genetic and environmental risk factors, and their interaction in schizophrenia.

## 1. Introduction

Schizophrenia is a psychotic illness characterised by multifaceted psychopathology and dysfunction [1–5], with a European prevalence estimate for psychotic disorders (including schizophrenia) of 1.2% [6, 7]. This debilitating disorder is characterised by heterogeneous display of positive symptoms (hallucinations, delusions, and thought disorder), negative symptoms (avolition, restricted affect, poverty of speech, and social withdrawal), and cognitive dysfunction (e.g., working memory deficits, executive function, and attentional dysfunction), which typically emerge during late adolescence and young adulthood. Antipsychotic drugs which are currently available and commonly prescribed are efficacious against positive symptoms including hallucinations and delusions but are associated with significant side effects which negatively impact on compliance [4, 8], have little beneficial effect against the negative or cognitive symptoms, and moreover are not effective in all patients [9].

Schizophrenia is also a highly heritable disorder of neurodevelopment, where the development and expression of positive or psychotic symptoms are best viewed as signifying the outcome of a pathobiological cascade which originates in early brain development [4, 10]. Research over the past decade has significantly advanced our understanding of the genetic basis of schizophrenia, identifying risk loci, and suggesting biologically plausible mechanisms by which genetic risk is conferred [11], but much is still unknown [9]. A multitude of factors including, but not restricted to, gene × environment (G × E) and gene × gene (G × G) interactions, epigenetic modifications, and considerable heterogeneity at a genetic and phenotypic level, complicate our understanding of the role of these genes in the disorder and the translation of genetic advances into novel biological treatment targets [12, 13]. G × E interactions in schizophrenia might reflect genetic control of responses to protective or adverse environmental factors, as well as context-dependent phenotypic expression. However, recent articles have highlighted the challenges associated with selecting appropriate statistical methods for identifying G × E interactions in schizophrenia and other neuropsychiatric disorders [14, 15]

Adoption and twin studies have confirmed that schizophrenia has a significant heritability component [16] with risk to develop schizophrenia or a related psychotic disorder positively correlated with degree of genetic similarity [17]. However, twin studies conducted in schizophrenic patients indicate that genes contribute no more than 50% to aetiology suggesting that developmental and environmental factors also have a major role to play [9]. Epidemiological studies have suggested that a diversity of factors including prenatal infection/immune activation, paternal age, malnutrition, hypoxia-related obstetric complications, and childhood/adolescence social stress and cannabis abuse are associated with increased risk for development of this disorder [2]. A “multihit” model has been proposed, and two crucial time windows associated with early brain development and maturation during adolescence have been identified as particularly sensitive periods for exposure to adverse environmental events, which could eventually trigger schizophrenia-relevant biological sequelae [18].

Recent genomewide association study (GWAS) analyses have identified multiple common schizophrenia risk alleles, each contributing a small effect, although they have provided mixed support for some of the more prominent common risk alleles identified in case-control and family-based genetic association studies [5, 19, 20]. Additionally, the discovery of microRNA changes and copy number variations (CNVs) in schizophrenia highlight the contribution and impact of rare and highly penetrant alleles in conferring genetic risk for schizophrenia [21, 22]. If a multiple-hit hypothesis is in fact an underlying model for the majority of cases for schizophrenia, it is likely to involve a combination of single nucleotide polymorphisms (SNPs), rare penetrant mutations, and environmental factors [23]. A large number of G × E interaction studies in patients with schizophrenia have focused on one candidate gene interacting with a specific environmental exposure. Since these studies have a specific prior hypothesis, they can be investigated with a modest sample size. Recent reviews of G × E interactions across clinical and preclinical studies in schizophrenia have however highlighted the relative paucity of relevant clinical data, noting that several of the animal models discussed in the present review have consequently selected G × E manipulations based on either combinations of genetic and environmental factors which have been (a) independently associated with schizophrenia, but not in combination, and/or (b) target common biological pathways implicated in schizophrenia, for example, disturbance of dopaminergic (DA) transmission [24–26].

G × E interactions in schizophrenia may also take the form of environmental factors impacting on DNA methylation, producing changes in gene expression through epimutations [27, 28]. Epigenetic factors represent an important mechanism whereby the adverse effects of environmental risk factors may impact gene expression. This topic has previously been discussed in more detail in relation to schizophrenia (e.g., [27]) and genetic models of schizophrenia [28]. One notable example is advanced paternal age (APA), which has been shown to be a risk factor for schizophrenia [29] as well as a host of other adverse neurodevelopmental outcomes (attention deficit hyperactivity disorder (ADHD), 30; autistic spectrum disorder (ASD), [30]). The predominant hypothesis in the field postulates age-related accumulation of* de novo* mutations in paternal sperm DNA [31], with a growing body of evidence suggesting that epigenetic changes in these cells could also be implicated [32, 33].

The present review will seek to summarise recent research which has been conducted on modelling of G × E interactions in schizophrenia using preclinical genetic models, primarily constitutive knockout or transgenic lines. There will be an emphasis placed on summarising evidence for psychosis-relevant features in the models, together with any evidence for mechanistic-based interrogation of the underlying pathophysiology.

## 2. Genetic Basis of Schizophrenia

Meta-analyses of twin and adoption studies have shown that heritability accounts for approximately 70% of disease risk in schizophrenia [34], where the magnitude of risk varies widely, from relatively modest odds for common genetic variants to substantial risks due to rare variants. Rare chromosomal deletions and duplications can increase risk for the disorder, with the magnitude of the increase in risk substantially greater than that observed for common variants [35–37].

GWAS data has implicated several candidate genes with a strong link to the pathophysiology of the disorder, while questioning the impact of hitherto prominent susceptibility targets (e.g.,* disrupted-in-schizophrenia-1 *(*DISC1*),* neuregulin-1* (*NRG1*)) [38]. The most recent analysis has identified 108 agreed loci that contribute to risk for schizophrenia; specifically, the Psychiatric Genomics Consortium (PGC) collaborative molecular genetic study of almost 37,000 patients with schizophrenia and 113,000 healthy controls identified 83 novel risk markers and replicated 25 existing markers [39]. The study pointed particularly to genes involved in neurodevelopment, the immune and stress response, glutamatergic neurotransmission, and DA D2 receptor activity.

CNV analyses which detect structural variants in the form of submicroscopic deletions and duplications of DNA have identified rare* de novo* and inherited variants that confer high risk for schizophrenia (Odds Ratio = 3–20) [40]. An exome-sequencing study involving 2536 schizophrenia cases and 2543 controls demonstrated a polygenic burden primarily arising from rare (less than 1 in 10,000), disruptive mutations distributed across many genes [41]. These authors were able to detect several small and highly enriched sets, notably of genes related to N-methyl-D-aspartate (NMDA) receptor-associated postsynaptic density-95 (PSD-95) protein complexes, activity-regulated cytoskeleton- (ARC-) associated interacting proteins and fragile × mental retardation protein (FMRP) targets [42].

Importantly, some of the genetic factors linked with increased risk for schizophrenia also display association to broader phenotypes including bipolar disorder, as well as major depression, ADHD, and autism [43], suggesting that clinical overlap between these disorders may in part reflect a shared genetic basis. In a recent combined GWAS of 19779 bipolar disorder and schizophrenia cases versus 19423 controls, in addition to a direct comparison GWAS of 7129 schizophrenia cases versus 9252 bipolar disorder cases, the authors identify five previously identified regions reaching genome-wide significance as well as a novel locus [44]. These authors reported a significant correlation between a bipolar disorder polygenic risk score and the clinical dimension of mania in patients with schizophrenia. Overlapping disease pathways may, in part, explain shared symptoms across diagnoses, as well as multiple diagnoses within patients [45].

## 3. Mutant Models of G × E Interactions in Schizophrenia

Interactions between genetic risk and environmental stressors at various stages of life appear important in the development of schizophrenia [46–48]. Preclinical genetic models provide tools for assessing the relative contribution of genes, exposure to environmental pathogens, and their interaction, on the development of schizophrenia-relevant phenotypes [25, 48, 49]. Preclinical modelling of G × E interactions related to schizophrenia has typically involved examining the phenotypic consequences of epidemiologically relevant but also translationally valid, experimental manipulations in various candidate risk gene mutant models [50, 51]. Combining an environmental challenge with a genetic mutation can produce both protective and adverse effects. It has been noted that the potential to generate such results should be incorporated within the study design and that exclusively focusing on a limited set of prespecified outcome measures may exclude the possibility of reporting such unexpected and complex bidirectional results [28]. Particularly in the context of evidence for a shared genetic basis underlying several major neuropsychiatric disorders, the discovery of novel behavioural phenotypes in preclinical models of G × E interactions has the potential to inform us about the role of the environment in evoking diverse clinical outcomes in patients with the same mutation.

Timing of the environmental insult is an important factor that needs to be considered during the development and evaluation of the G × E model. Mutant modelling of G × E interactions in schizophrenia studies has typically involved environmental manipulations at particular periods of brain development (e.g., early pregnancy or adolescence) which are regarded as important to the pathogenesis of schizophrenia. These critical periods of brain development correspond to early life (pre-, peri-, and early postnatal period) or later (adolescent) stages in humans [52, 53].

While many of the studies discussed below, which aim to simulate G × E interactions implicated in psychosis in rodent models, consist mostly of descriptive analyses, a growing number of studies are starting to provide important mechanistic insight into the molecular/cellular basis underlying such interactions. Elucidating the biological mechanisms underlying synergistic G × E effects on emergence of neuropsychiatric phenotypes necessitates interrogation of the molecular basis of the observed phenotypes.

## 4. Modelling Schizophrenia in Rodents

While it is impossible to model schizophrenia* per se *in mice or other rodents, three important criteria need to be satisfied in order for any experimental model to claim validity for the disorder. Firstly, the model should reflect, at least in part, the etiopathological basis of the disorder. Secondly, while research has emphasized the neurodevelopmental aspect of schizophrenia, its clinical onset is postpubertal. This fact emphasises the importance of examining the data of young animals as part of any G × E interaction modelling effort, so that the trajectory from insult during early development or young adulthood to the emergence of adult phenotypes can be established. Thirdly, the experimental model should reflect endophenotypes relevant to schizophrenia in adulthood. Endophenotypes are quantifiable, intermediate disease features that bridge the gap between the overt manifestations of schizophrenia and underlying risk genes [54]. Earlier reviews have highlighted the value of utilising endophenotypic endpoints in preclinical genetic studies, where intermediate biological or behavioural phenotypes are less susceptible to confounding influences and are therefore easier to investigate [26]. Schizophrenia-relevant endophenotypes include behavioural deficits (e.g., working memory impairment, deficits in sensory or sensorimotor gating, and social withdrawal) and several histological/structural changes such as enlarged lateral ventricles and deficits in a specific subtype of interneurons in the cortex.

Recently, efforts have been made to identify equivalent behavioural domains and functional assays between humans and animals, including the Measurement and Treatment Research to Improve Cognition in Schizophrenia (MATRICS) and Cognitive Neuroscience Treatment Research to Improve Cognition in Schizophrenia (CNTRICS). More recently, the Research Domain Criteria (RDoC) initiative from the National Institute of Mental Health (NIMH) aims to reclassify psychiatric disorders according to basic dimensions of functioning, where each behavioural domain is studied across multiple levels of analysis, from genes to neural circuits to behaviour in both animal models and humans, assuming that these behavioural domains share more or less similar underlying mechanism across species [55]. RDoC includes the following domains: negative valence systems (fear, anxiety, and loss), positive valence system (reward learning, reward evaluation), cognitive systems (attention, perception, working memory, and cognitive control), systems for social processes (attachment formation, social communication, perception of self, and perception of others), and arousal/modulatory systems (arousal, circadian rhythms, sleep, and wakefulness).

## 5. Modelling Environmental Risk Factors Relevant to Schizophrenia in Rodents

There is a general consensus among schizophrenia researchers that diverse biological, environmental, and psychosocial insults, across the lifespan, accumulate in their adverse impact on an already developmentally compromised brain to result in the development of psychotic illness [26, 48]. Consistent with the well-considered “stress-vulnerability” aetiological model, these extend from early biological and psychosocial insults during the prenatal or perinatal period (including winter birth, maternal infections or immune challenge, and other obstetric complications [2, 48]), through exposure to adversity during infancy and childhood (e.g. societal factors, childhood abuse; [56]), to pathogenic factors present during adolescence and young adulthood (exposure to psychosocial stressors, prolonged exposure to drugs of abuse including cannabis [57]). As noted above in relation to genetic factors, numerous environmental factors associated with schizophrenia and other psychotic disorders are also associated with a range of other neurodevelopmental and neuropsychiatric outcomes, including autistic spectrum disorder, attention-deficit hyperactivity disorder, and epilepsy [20], leading some authors to propose that schizophrenia is best conceptualised as one of a spectrum of clinical outcomes that result from exposure to selected genetic or environmental factors, or both [20]. Translational efforts to model such factors on rodents have generally sought to develop ethologically appropriate (e.g., maternal deprivation, postweaning social isolation, or social defeat during adolescence to study the effects of psychosocial stress on neurobehavioural measures across development in mice/rats) or practicable and exposure-relevant biological manipulations (e.g., inflammatory responses after infection and cytokine-mediated effects on brain development using polyinosinic-polycytidylic acid (Poly I:C) and lipopolysaccharide (LPS) in rats/mice) to investigate the biological underpinnings of G × E interactions.

## 6. Infection and Schizophrenia

It is well established that prenatal influenza exposure is associated with increased risk of developing schizophrenia in the offspring [58, 59]. This risk liability has been shown to extend to other viral and bacterial agents, as well as exposure to parasitic agents such as* Toxoplasma gondii* [2, 60]. The emergence of schizophrenic symptomatology in adult offspring has been shown to be dependent upon maternal infection at different gestational points throughout pregnancy [61, 62], which is an important consideration when developing valid animal models of maternal infection in schizophrenia. While a multitude of infectious agents have been associated with increased risk for schizophrenia, it is proposed that the common pathophysiological mechanism underlying their “schizophregenicity” involves activation of the maternal immune system [2, 62].

Preclinical experimental models have been developed which involve prenatal exposure to infection, immune activation, or another relevant biological insult. These models have included gestational exposure to human influenza virus, the bacterial endotoxin LPS, and Poly I:C, a synthetic analogue of double-stranded RNA which is recognized as an infectious pathogen by the human immune system [63]. In the rodent prenatal Poly I:C model, administration of Poly I:C to pregnant dams causes elevations in maternal serum cytokines that are accompanied by emergence in adulthood of behavioural and neural phenotypes related to those evident in schizophrenia [64]. Timing of immune challenge is a significant determinant of brain and behaviour outcomes in subsequent offspring. It has been shown that the effects of maternal immune challenge during gestation between early (gestational day [GD] [9]) and late (GD17) pregnancy periods in mice are dissociable in terms of foetal brain cytokine responses to maternal inflammation and subsequent functional effects [65, 66]. These challenge periods correspond to the end of the first trimester (GD9) and middle/late phase of the second trimester (GD17) in humans [67, 68].

Poly I:C treatment during early pregnancy is associated with schizophrenia-related endophenotypes in adult offspring including deficits in prepulse inhibition (PPI [67, 68]) as well as latent inhibition (LI [69]), two measures of preattentional and selective attention processes, respectively, which are disturbed in schizophrenia. Across various measures of social interaction, both early and late gestational treatment Poly I:C in dams has been shown to disrupt sociability and social cognition [66, 70, 71]. Similarly, offspring of Poly I:C-treated dams display a hyperexploratory phenotype in a novel environment [64], as well as increased behavioural sensitivity to DA agonists and NMDA receptor antagonists [72, 73]; both of these features are considered proxy measures for the positive symptoms of schizophrenia. Structural brain endophenotypes associated with schizophrenia have also been demonstrated in the brains of adult offspring of Poly I:C treated mice; these include lateral ventricular enlargement and decreased hippocampal volume [74, 75].

As the majority of individuals exposed to neurodevelopmental insults such as infections do not develop schizophrenia in adulthood, it is important to assess the additive and interactive effects of infection and genetic vulnerability on the development of schizophrenia-relevant endophenotypes.

### 6.1. NRG1 × Immune Challenge

*Neuregulin-1* (*NRG1*) is putative risk gene which has been widely studied in relation to its association with schizophrenia [76–78]. In meta-analysis, the association between the* NRG1* schizophrenia-associated risk haplotype (HapICE, first reported by Stefansson et al. [76]) and schizophrenia has proved replicable [77]. NRG1 belongs to a family of growth factors which are encoded by four genes (*NRG1-4*); it has greater than 30 isoforms, grouped into six “types” (I–VI) that are differentiated on the basis of N-terminal sequence, expression of the *α* or *β* epidermal growth factor- (EGF-) like domain, and presence of a transmembrane (TM) region [79, 80]. NRG1 proteins are ligands for ErbB receptor tyrosine kinases; this, in turn, activates intracellular signalling pathways that are known to play a prominent role in diverse developmental processes implicated in schizophrenia [79, 80]. NRG1 is expressed in diverse brain areas, including the PFC, hippocampus, cerebellum, and substantia nigra in both humans and rodents [80]. NRG1 isoforms differ in domain structure and expression levels in various tissues/cells during brain development and, later, in adulthood; isoform-specific roles and properties, particularly in relation to the NRG1-schizophrenia association, remain poorly understood [80]. This level of genetic complexity highlights the difficulty associated with generating accurate preclinical genetic models of NRG1 dysfunction in schizophrenia. Clinical genetic analyses have supported the association between* NRG1* variation, inflammatory function, and neurogenesis. Interaction between the genes encoding the proinflammatory cytokine interleukin 1*β* (IL-1*β*) and* NRG1* genotype increases the risk of schizophrenia and shortens the age of onset for the disorder [81]. Additionally, a missense mutation in* NRG1* has been reported to increase activation of proinflammatory cytokines such as interleukin 6 (IL-6), tumor necrosis factor *α* (TNF-*α*), and interleukin 8 (IL-8) in patients with schizophrenia [82].

Various* NRG1* knockout and transgenic mouse lines have been developed to study the relationship between altered NRG1 signalling and impact on behavioural and brain endophenotypes relevant to schizophrenia [26, 83]. Mice with heterozygous knockout of the transmembrane- (TM-) domain truncation of exon 11* NRG1*, which is associated with the disruption of several* NRG1* splice variants, display increased novelty-induced hyperactivity, which is reversed by antipsychotic treatment [76, 84, 85]. Disruption to PPI has also been reported in the TM-domain* NRG1* mutant line [86–88], and they also display deficits in social interaction [85].

Comparisons with alternative TM-domain or more isoform-specific* NRG1* deletions indicates that differences in the targeting strategy, as it relates to the* NRG1* gene, can produce very different effects across various neurobehavioural measures. For example, in contrast with the exon 11 TM-domain lines, mutant mice with targeted disruption of type I/type II* NRG1* do not show a hyperactive phenotype [89, 90]. Similarly, no significant behavioural impairments, aside from mild cognitive deficits, were observed in a TM-domain mutant line with a truncation from exon 9 [91].

O'Leary et al. [92] examined the unique and combined effects of prenatal immune challenge (*via* administration of Poly I:C at GD9) and postnatal cross-fostering (a control procedure which can also act as a stressor, where offspring are separated from dams and raised by surrogate mothers) in mice with partial TM-domain (exon 9) deletion of* NRG1*. In this study, distinct phenotypic effects across schizophrenia-related behavioural measures (social interaction, PPI, and open-field exploration) were observed for both individual environmental variables as well as interactions between these factors and genotype [92].* NRG1 *mutants demonstrated impaired social novelty preference, PPI, and a sex-specific (females only) decrease in spatial working memory performance, irrespective of exposure to the stressor. Poly I:C treatment also disrupted PPI and working memory performance across both genotypes. Combining* NRG1 *disruption and prenatal immune challenge caused deficits in social behaviour and spatial working memory, whereas combining* NRG1* disruption with the early life stressor (cross-fostering) impaired social novelty preference, a measure of social cognition. No synergistic effect of* NRG1* disruption and prenatal immune challenge was observed in relation to PPI, which may be attributable to a masking effect of* NRG1*-related PPI disruption on potential* NRG1* × prenatal immune challenge interactions on sensorimotor gating. However, the combination of prenatal immune challenge and cross-fostering (i.e., E × E) also produced several behavioural deficits in the open field, social behaviour, and PPI. The results of this study suggest that the emergence of schizophrenia-relevant endophenotypes can arise from multiple, often very complicated, interactions involving individual genes interacting with several biological and psychosocial factors.

### 6.2. DISC1 × Immune Challenge

*DISC1* is a prominent schizophrenia risk gene, which was originally identified at the breakpoint of a balanced chromosomal translocation cosegregating with mental disorders in a large Scottish kindred [93]. Subsequent clinical genetic studies have identified evidence for involvement of common and rare risk variants at this locus in the etiology of a range of neuropsychiatric disorders, including schizophrenia, schizoaffective disorder, bipolar disorder, and recurrent depressive disorder [94, 95]. DISC1 is an essential synaptic protein, which interacts with a wider molecular network to mediate processes associated with cellular and synaptic function [96]. Mutant models of* DISC1* gene function display anatomical, behavioural, and pharmacological phenotypes relevant to several neuropsychiatric disorders, including schizophrenia and depression [97–103]. As with the* NRG1* mutant data, these* DISC1* mutant phenotypic analyses again illustrate how different mutations in the same gene can result in divergent phenotypic outcomes. For example, a transgenic line with inducible and reversible expression of a DISC1 C-terminal fragment under the calcium/calmodulin-dependent protein kinase II alpha (*α*CAMKII) promoter demonstrated impaired social functioning and disruption of spatial working memory [99]. In a transgenic line with expression of a dominant-negative truncated form of DISC1 under the *α*CaMKII promoter, mutants exhibited novelty-induced hyperactivity but no other major phenotypes [98]. Double transgenic mice expressing human* DISC1* under the cytomegalovirus (CMV) promoter with tetracycline under the *α*CaMKII promoter showed a hyperactive phenotype, as well as deficits in social interaction and spatial memory [101]. Another group described two mouse line carrying point mutations in* DISC1* (L100P and Q31L), where abnormalities associated with schizophrenia were observed in the L100P line; these included deficits in PPI and LI, as well as working memory, many of which were shown to be reversible by antipsychotic administration [97].

Employing the Poly I:C immune challenge procedure, Lipina et al. [104] demonstrated that the mutant offspring of L100P dams who had been given a single injection of Poly I:C on GD9 demonstrated more prominent PPI and LI deficits, as well as impaired working memory and sociability, relative to L100P controls or both challenged and unchallenged wildtype controls, where moderate deficits in these tasks were already observed following the genetic or environmental manipulation alone. Coadministration of an IL-6 antagonist blocked the disruptive effects of prenatal Poly I:C on PPI and LI performance in L100P mice, providing a direct link between Poly I:C treatment and behavioural disruption in these mice.

The phenotypic effects of combining prenatal immune challenge with* DISC1 *disruption were also described in a study conducted in mice with inducible expression of mutant* hDISC1* in forebrain neurons [50, 101]. Poly I:C treatment increased anxiety in mutants and controls in the open field, and both challenged and nonchallenged* DISC1* mutants displayed lateral ventricular enlargement relative to controls. Male* DISC1* mutant offspring of dams treated with Poly I:C at GD9 demonstrated decreased social approach behaviours, as well as an anxiogenic phenotype (less time in the open arms of the elevated plus maze) and depression-like behaviours (i.e., decreased latency to immobility in the forced swim test). These behavioural deficits were accompanied by altered serotonergic neurotransmission in the hippocampus, decreased hypothalamic-pituitary-adrenal (HPA) axis reactivity and attenuation of genotypic enlargement of the lateral ventricles, as well as differential modulation of secretion of inflammatory cytokines [50].

Another study examined the interaction of* DISC1* mutation with* neonatal *treatment with Poly I:C between postnatal days 2 and 6 [105]. While neither the* DISC1* mutation nor neonatal immune challenge were independently associated with any phenotypic effects, transgenic mice expressing a dominant-negative form of* DISC1* displayed a pronounced schizophrenia-related phenotype across several cognitive endophenotypes (spontaneous Y-maze alternation [which measures working memory processes], recognition memory, and contextual fear memory) following neonatal immune challenge. Social interaction and MK-801-induced hyperactivity were also selectively altered in Poly I:C-treated* DISC1* mutants. These behavioural deficits were accompanied by a decrease in parvalbumin-positive interneurons in the medial prefrontal cortex (a cellular endophenotype for schizophrenia) of* DISC1 *× neonatal immune challenge mutants. It was later shown, employing the same experimental design, that the antipsychotic drug clozapine successfully reversed the recognition memory deficits in* DISC1 *mutants exposed to neonatal Poly I:C [106].

A recent study examined the interaction between* DISC1* genotype, employing the transgenic model of inducible expression of dominant-negative mutant human* DISC1*, and prenatal exposure to the toxin lead (Pb2+), to assess the development of neuropsychiatric phenotypes in resultant lead-exposed offspring [107]. Lead exposure was associated with the expression of increased anxiety, disruption of PPI, increased responsivity to the NMDA receptor antagonist MK-801, and ventricular enlargement (also observed in nonstressed* DISC1 *mutants versus controls). The authors reported several, often sex-specific, synergistic effects, demonstrating more pronounced PPI deficits, heightened MK-801 responsivity, and alterations in exploratory activity and ventricular volume in* DISC1* mice exposed to lead.

### 6.3. Nurr1 × Immune Challenge

Nurr1 is a member of the orphan steroid hormone receptor family which is involved in key processes including differentiation, migration, and survival of midbrain DA neurons [108], as well as regulation of the expression of genes which are crucial for DA neurotransmission [109]. The combination of partial knockout of* Nurr1* and prenatal immune activation* via* late gestational Poly I:C administration resulted in additive effects on locomotor hyperactivity in a novel environment and PPI disruption, where deficits across both measures were already observed following genetic disruption of* Nurr1 *or exposure to Poly I:C alone. In contrast, multiplicative disruptive effects of both genetic and environmental manipulations were observed for measures of attentional function including LI persistence and a measure of sustained attention [110]. Synergistic interactions between* Nurr1* haploinsufficiency and prenatal immune activation on DA D2 receptor density in the nucleus accumbens core and shell were also reported, as well as a significant decrease and increase in tyrosine hydroxylase and catechol-O-methyltransferase (COMT) density, respectively, in the medial prefrontal cortex [110].

## 7. Cannabis Use and Schizophrenia

Recent epidemiological surveys have calculated mean estimates of lifetime prevalence of cannabis use of 25% and 35.8% among youth aged 15-16 in the UK and USA, respectively [111, 112]. Therefore, a significant number of young people are exposed to cannabis during an important neurodevelopmental stage characterised by maturation of neural circuitry across several brain areas implicated in schizophrenia and other neuropsychiatric disorders. Lifetime cannabis use increases risk for developing a psychotic disorder [113, 114], where the risk quotient is highest among individuals who use cannabis during adolescence [115–118]. However, despite high prevalence estimates for lifetime cannabis use, a relatively small proportion of cannabis users go on to develop subclinical symptoms or a clinical psychotic disorder [119]. This may be explained by the interaction between cannabis-related psychosis risk and genetic disposition, as well as the copresence of other adverse environmental conditions [120]. It may also reflect differential concentrations of delta-9-tetrahydrocannabinol (THC) and cannabidiol in cannabis products. THC is the principal psychotomimetic ingredient of cannabis; cannabidiol, in contrast, is a cannabinoid which can exert anxiolytic and potentially antipsychotic effects [117, 119].

A recent analysis, conducted in a population-based sample, revealed a negative association between cannabis use in early adolescence and cortical thickness (a morphological endophenotype for schizophrenia) in male adolescents with a high genetic risk for schizophrenia, as indicated by their risk profiles across 108 genetic loci identified by the Psychiatric Genomics Consortium in a large genome-wide comparison of patients with schizophrenia and control individuals [121]. G × E studies examining the link between cannabis and psychosis in humans face the challenge of conclusively excluding the possibility that individuals with a particular genotype or profile of exposure to environmental adversity may be more likely to use cannabis, as opposed to cannabis exposure independently affecting the pathway to psychosis [122]. Delta-9-tetrahydrocannabinol (THC) is the principal psychotomimetic ingredient of cannabis; cannabidiol, in contrast, is another component of cannabis which is thought to exert anxiolytic effects [123]. Prolonged exposure to THC during the period corresponding to adolescence in rats and mice is associated with the emergence of deficits across several schizophrenia-related endophenotypes, including attentional and memory function (PPI, recognition memory), novelty-induced hyperactivity [123], and deterioration in reinforcement learning performance [124]. It is also accompanied by neuronal hyperactivity in the mesocorticolimbic DA pathway as well as modification of prefrontal cortical molecular pathways [125]. Cannabinoid modulation of activity of DA projections from the brain stem to the striatum, in particular, has been linked with the development of cannabis-induced psychosis [126].

### 7.1. NRG1 × Cannabis Exposure during Adolescence

A putative association between* NRG1* genotype and cannabis-related psychosis has not yet been examined in clinical samples. A genome-wide linkage scan, and follow-up association analysis, for cannabis dependence in African-American and European-American families, revealed that* NRG1* variation was associated with increased risk for cannabis dependence in African-Americans, and this effect was pronounced in females [127].

Male TM-domain* NRG1* mutant mice have shown increased susceptibility to several of the neurobehavioural effects of acute THC relative to wildtype controls. These genotypic effects have included greater sensitivity to the PPI-enhancing and anxiogenic effects of THC, as well as its locomotor activity suppressing effects [128–130]. These authors also observed that THC-induced increase in immediate early gene (*c-fos*) expression was greater in the shell of the nucleus accumbens, central nucleus of the amygdala, paraventricular nucleus, and dorsolateral bed nucleus of the stria terminalis of* TM-NRG1* mutants relative to controls [129]. Adding complexity to the interpretation of G × E effects in this model and suggesting the presence of second-level E × E interactions, this genotype-dependent increase in* c-fos* expression was only observed in mice who had been subjected to behavioural assessments. In a complementary manner,* TM-NRG1* mutants also demonstrated increased tolerance to the locomotor suppressant and anxiogenic effects of the synthetic cannabinoid CP 55,940, administered during adulthood [131].* TM-NRG1* mutants were also resistant to the cannabinoid-induced decrease in investigative social behaviours compared to controls [132]. The latter study also showed that several of adolescent THC effects on cannabinoid receptor 1 (CB1R) and 5-HT2A receptor binding (decreased in* TM-NRG1* mutants, increased in wildtypes) in the substantia nigra and insular cortex were genotype-dependent. Adolescent THC also selectively increased NMDA receptor binding in the auditory cortex, cingulate cortex, and hippocampus of* TM-NRG1* mutants [132], as well as inducing differential expression of proteins implicated in NMDA receptor trafficking and glutamatergic function in the hippocampus of adolescent THC-treated* TM-NRG1* mutants versus controls [133].

Cannabidiol is another psychoactive component of cannabis which has been reported to possess anxiolytic [134] and putative antipsychotic properties [135]. Long and colleagues examined the neurobehavioural effects of chronic cannabidiol during adulthood in TM-NRG1 mutants relative to controls [136]. Chronic cannabidiol selectively enhanced social interaction and increased GABA_A_ receptor binding in the granular retrosplenial cortex in* TM-NRG1 *mutants but had no effect on PPI or novelty-induced exploratory activity [136]. Collectively, studies conducted on THC, synthetic cannabinoid, and cannabidiol effects in TM-domain* NRG1* mutants would indicate altered sensitivity to the neurobehavioural effects of this class of drugs, in a manner which is dependent upon timing and duration of treatment.

### 7.2. DISC1 × Cannabis Exposure during Adolescence

A recent study investigated the interaction, at a preclinical level, between mutation in* DISC1 *and the effects of chronic adolescent administration of THC [137]. In this model, a putative dominant-negative form of* DISC1* (*DN-DISC1*) which is expressed under the control of the alpha-CAMKII promoter in forebrain pyramidal neurons, chronic treatment with THC during adolescence (postnatal days 28–48) worsened deficits in cue-dependent fear memory in* DN-DISC1 *mice, while neuronal activation induced by fear memory retrieval was also selectively impaired in* DN-DISC1* mice.* DN-DISC1* mice also demonstrated deficits in contextual fear memory irrespective of treatment condition. The combinatorial effect of adolescent THC exposure and* DN-DISC1* expression on the endocannabinoid system was also indicated by a synergistic reduction in synaptic CB1R expression in the prefrontal cortex, hippocampus, and amygdala.

### 7.3. COMT × Cannabis Exposure during Adolescence

COMT is an enzyme involved in the catabolism of catecholamines and is the principal enzyme controlling the metabolism of DA in the prefrontal cortex [138]. A common functional polymorphism in the* COMT *gene, the Val158Met variant, has been associated with differential reactivity to stressful stimuli. Individuals with the* COMT* Val/Val (high enzyme activity) genotype exhibit decreased affective reactivity to stress relative to carriers of Met/Met, the low enzyme activity allele [139]. Studies have shown that the disruptive effects of childhood abuse on adult emergence of cognitive deficits [140] and frequency of self-reported psychotic experiences [141] are present only in* COMT* Met/Met carriers. In one of the first clinical G × E reports reported for schizophrenia, risk to develop psychosis was shown to be highest among those who used cannabis during adolescence and were* COMT* Val/Val carriers [142]. Preclinical genetic studies employing a constitutive* COMT* gene knockout model, which looked at the interaction between chronic intermittent THC and Win 55,212 (a synthetic CB1R agonist) exposure during adolescence and* COMT* deletion, demonstrated that* COMT* genotype modulated responsivity to adolescent cannabinoid effects in relation to hyperactivity in a novel environment, working memory, and PPI [123, 143]. Specifically, THC treatment reversed enhancement of working memory in* COMT* knockout mice and produced changes in exploratory activity and PPI that were not observed following COMT knockout or THC treatment alone These deficits were accompanied in a genotype-dependent manner by changes across morphological measures of DA-ergic and GABA-ergic function [144].

## 8. Social Stress and Schizophrenia

Exposure to psychosocial stressors, particularly at developmentally important time points, has been shown to both play a role in the development of a psychotic disorder and precipitate the onset of psychotic illness when the stressful experience occurs closer to the onset of the disorder [144–147]. One particular social stressor which has been both linked with increased risk for schizophrenia and modelled in preclinical assays is social defeat, which refers to the defeated feeling of subordination which is experienced following an adverse social encounter [148, 149]. Animals studies have consistently shown that exposure to social defeat is associated with changes across several schizophrenia-related endophenotypes, as well as HPA axis function, and corticolimbic DA neurotransmission (see [150] for detailed review of evidence). Generally, rats or mice subjected to social defeat demonstrate impaired social behaviour, as well as increased behavioural signs of anxiety and depression [151, 152].

### 8.1. NRG1 × Social Stress

The combined effect of* NRG1* heterozygous knockout and chronic social defeat stress (via intermittent access to an aggressive CD1 strain conspecific) during adolescence produced genotype-dependent working memory deficits and elevated basal cytokine levels during adulthood in* TM-NRG1* mutant mice relative to controls [86].* TM-NRG1* mutants displayed a genotypic increase in novelty-induced activity, disruption of PPI and social novelty preference, and decreased anxiety relative to wildtypes. However, the combination of repeated social defeat stress and partial* NRG1* knockout produced deficits in the Y-maze spontaneous alternation task (a measure of working memory), which were not observed in stressed wildtype controls. In contrast, in the sucrose preference test (a measure which is utilised to model anhedonia in rodents), stressed control mice displayed reduced sucrose preference (i.e., an “anhedonic” profile), whereas no such effect was observed in stressed* NRG1* mutants. Another recent study which compared the effects of acute and chronic exposure to a nonsocial stressor, restraint stress, during adolescence in* TM-NRG1* mutants versus controls reported increased sensitivity to the anxiogenic effects of acute stress exposure in mutants [153]. Chronic intermittent stress during adolescence also produced deficits in PPI in* NRG1* mutants relative to both stressed wildtypes and nonstressed mice belonging to both genotypes.* NRG1* mutants also demonstrated decreased corticosterone levels, as well as increased apical dendritic spine density and decreased apical dendritic lengths and complexity in layer II/III pyramidal neurons of the medial prefrontal cortex, following chronic restraint stress.

### 8.2. DISC1 × Social Stress

The phenotypic effects of social defeat stress during adulthood in mice were examined in DISC1 L100P and Q31L (a DISC1 line which demonstrates more affective disorder-related phenotypes and fewer psychosis-relevant phenotypes than the L100P line) mutants [154]. They reported decreased vertical activity levels during exploration in a novel environment, as well as social interaction in mice with heterozygous mutation in* DISC1* (L100P) following exposure to social defeat. While L100P mice displayed a deficit in PPI, and both L100P and Q31L mice displayed disruptions in LI, social defeat did not worsen deficits in these tasks for any group. Social defeat stress during adulthood was also associated with increased immobility in the forced swim test, as well as an anhedonic profile in the sucrose consumption test, but these effects were not genotype-dependent.

Another study employed the C′-truncated* DN-DISC1* model, where expression is under the control of the widely expressed prion protein promoter. Mutants and controls were subjected to three weeks of social isolation during middle and late adolescence (postnatal days 35–56). This manipulation resulted in the emergence of schizophrenia-related behavioural deficits, including PPI disruption, increased immobility in a forced swim test (a measure of behavioural despair which has been used to model apathy), and increased methamphetamine-induced locomotion, in mutants relative to isolated wildtypes and nonisolated mice of both genotypes [155].* DN-DISC1* × isolation mice also displayed decreased tyrosine hydroxylase expression, total tissue DA levels, and basal extracellular DA in the frontal cortex relative to all other genotype and environmental conditions. The same genotype-dependent effect of increased DA release was observed in the nucleus accumbens of isolated* DN-DISC1* mutants relative to all other groups. The observed behavioural and cellular endophenotypes were rescued by administration of the glucocorticoid receptor antagonist RU-486, suggesting that the heightened stress-induced corticosterone response in* DN-DISC1* × isolation mice might represent the mechanism underlying the schizophrenia-relevant behavioural and cellular phenotypes. A recent follow-up study which assessed DNA methylation of HPA-axis/glucocorticoid-related genes in the mesocortical DA-ergic neurons of* DN-DISC1* × isolation mice revealed altered DNA methylation of* tyrosine hydroxylase, brain-derived neurotrophic factor *(*BDNF*) and* FK506 binding protein 5* genes [53]; these epigenetic changes were once again reversed by glucocorticoid receptor antagonist treatment.

## 9. Other Genes Implicated in Pathogenesis of Schizophrenia: Evidence for G × E Interactions

### 9.1. Dystrobrevin Binding Protein 1 (DTNBP1)

Several studies have identified* DTNBP1* (or* dysbindin-1*) as a potential risk gene for schizophrenia [156–158]. Genetic association studies have shown that variations in this gene are associated with abnormal prefrontal cortical function in patients with schizophrenia, as well as episodic and working memory performance in healthy subjects [159–161]. The relevance of regionally specific loss of* DTNBP1* expression to the pathophysiology of this neurodevelopmental disorder is highlighted by postmortem studies revealing a decrease in* DTNBP1* expression in neurons of the dorsolateral prefrontal cortex and hippocampus [162, 163]. At a cellular level, DTNBP1 is mainly expressed in synaptic sites and plays an important role in synaptic homeostasis by regulating neurotransmitter vesicle exocytosis and vesicle biogenesis in neurons. DTNBP1 is also found in the nucleus, where it is reported to regulate transcription factor NF-kappa B activity to promote the expression of matrix metalloproteinase protein-9 (MMP-9), a matrix metalloproteinase that influences synaptic plasticity and learning and memory, and TNF-*α* [164]. In mice containing a loss-of-function mutation in* DTNBP1 *(sandy,* sdy*), they demonstrate hyperactivity, deficits in spatial learning and memory ability that are indicative of disrupted hippocampal function, and disruption of DA-ergic, glutamatergic, and GABA-ergic transmission in the prefrontal cortex [165–171]. While genetic background does appear to be an important factor in determining whether specific schizophrenia-related phenotypes are reported for the* sdy* mouse, memory impairment is a consistent phenotypic trait of DTNBP1-deficient mice irrespective of the mouse strain adopted [166, 172, 173].

Clinical studies provide some evidence indicating potentially significant associations between* DTNBP1* gene variation and the impact of adverse environmental risk factors on risk to develop schizophrenia [174, 175]. A study which examined potential interactions between* DTNBP1* variation and serious obstetric complications in a cohort of schizophrenia patients reported that the interaction of both factors influenced risk for schizophrenia [174]. It is also suggested that a common underlying molecular defect involving* DTNBP1 *contribution to the development of anxiety and stress-related disorders may involve changes in glutamatergic neurotransmission or DA-ergic function [175]. Indeed, characterisation of the behavioural phenotype of the* sdy* mouse revealed enhanced anxiety in these mutants, as indicated by a reduced habituation to novelty, reduced locomotor activity and time spent in the center of an open field test, and fewer open arm entries in the elevated plus maze test [166, 176]. It is possible, therefore, that* DTNBP1* mutation directly or indirectly affects neuronal circuitry subserving anxiety behaviours and stress responsivity, meriting further examination of potential interactions between stress-related environmental risk factors in schizophrenia and* DTNBP1 *gene abnormalities.

The timing of environmental insults during development and specific genetic vulnerability are important considerations in determining susceptibility to neurodevelopmental disorders and could differentially affect the degree to which* DTNBP1* mutations impact on structural and functional properties of neuronal cells, circuit connectivity, and overt behavioural phenotypes such as cognition, anxiety, and affective behaviour, leading to heterogeneous clinical phenotypes in schizophrenia [2, 25]. Evidence indicates that endogenous levels of the dysbindin protein in the mouse brain are higher during embryonic and early postnatal ages [177] suggesting adverse experiences during these vulnerable periods are more likely to affect the developmental course of dysbindin protein expression than those experienced during later stages of development. These findings highlight the critical nature of the temporal expression of* DTNBP1* in the brain and suggest that environmental factors experienced in early postnatal life and in adolescence may significantly impact on the trajectory of brain development and susceptibility to schizophrenia in those with* DTNBP1*-related genetic vulnerability.

### 9.2. SNAP-25

*Synaptosomal-associated protein of 25 kDa* (*SNAP-25*) is a gene associated with both synaptic transmission [178] and increased risk for schizophrenia [179, 180]. Mice containing a point mutation in the* SNAP-25* gene display several schizophrenia-associated endophenotypes including hyperactivity and increased behavioural sensitivity to psychostimulants, which are both mediated through DA D2 receptor activation [181, 182].* SNAP-25* mutants were demonstrated to be particularly sensitive to the disruptive effects of variable prenatal stress on social novelty preference [183]. In the same study, both the point mutation and variable prenatal stress independently produced disruption of PPI. In a recent study, prenatal exposure to nicotine throughout gestation and early perinatal development in mice with partial loss of function of* SNAP-25* resulted in increased hyperactivity, social interaction deficits, and deficits in long-term depression, which are paralleled by changes in the affinity of the DA D2 receptor [184].

### 9.3. BDNF

Brain-derived neurotrophic factor (BDNF) is implicated in diverse neurodevelopmental processes, including neuronal differentiation and survival, and plasticity, and may be important to the pathophysiology of schizophrenia [162, 185]. Theleritis et al. [186] demonstrated that* BDNF* genotype is related to childhood trauma but not to cognitive deficits in first episode schizophrenia. Exposure of pregnant mice to restraint stress was associated with increased* BDNF* expression in the frontal cortex and hippocampus of adult offspring [187]. A recent study evaluated the interaction between prolonged adolescent exposure to escalating doses of methamphetamine and heterozygous disruption of* BDNF* in mice and demonstrated that decreased* BDNF* expression may alter sensitivity to psychostimulant exposure at important developmental periods [188]. Methamphetamine-treated wild-type mice, but not* BDNF* heterozygous mice, showed locomotor sensitization to acute 3 mg/kg D-amphetamine, and this study also demonstrated increased sensitivity to amphetamine-induced disruption of PPI in* BDNF* heterozygotes [189].

### 9.4. RELN

Reelin is a protein that is involved in brain development and synaptic plasticity; Reelin-mediated signalling pathway dysfunction has been linked with the pathophysiology of schizophrenia [190, 191].* Reeler* is an autosomal recessive mutant mouse containing a mutation in the* RELN* gene, and several studies have examined the phenotypic consequences of interaction between early life adversity and the heterozygous* reeler* mouse phenotype. Interestingly,* reeler *mutants who were prenatally exposed to the neurotoxin chlorpyrifos [192] or early maternal separation [193] demonstrated a reversal of genotypic deficits across a number of schizophrenia-relevant endophenotypes; these included abnormalities in ultrasonic vocalisations and exploratory behaviour, as well as social interaction [193]. Neither chlorpyrifos exposure nor maternal separation alone exerted any effects on offspring behaviour. A recent study examined the phenotypic consequences of prenatal hypoxia on schizophrenia-related phenotypes in heterozygous* reeler *mice [194]. Exposure to prenatal hypoxia at embryonic day 17 (E17) was associated with a genotype-independent increase in anxiety (measured in the open-field test). No effect of genotype on PPI was observed, but a small treatment-related increase in PPI across both genotypes was reported [194].* RELN* genotype × prenatal hypoxia interaction was found in relation to frontal cortex volume, which was increased in wildtypes, but the genotypic increase in* RELN* mutants was decreased following prenatal hypoxia exposure. A selective reduction in glucocorticoid receptor protein levels in the hippocampus of stressed* RELN* mutants was also observed.

## 10. Discussion

The current review provides a summary of findings arising from the growing body of research on the generation of animal models of schizophrenia based on the interaction of genetic mutations and well-characterised environmental factors ([28, 49, 195]; see Tables 1 and 2 for summary of G × E findings related to selected schizophrenia-associated genes). These findings support the proposed “multihit” diathesis-stress model, whereby vulnerability to schizophrenia involves both the independent contribution and synergistic convergence of temporally sensitive biological and environmental factors across development. Identification of biological and environmental influences across critical developmental periods and the mechanistic basis for their interaction may eventually result in enhanced identification of schizophrenia risk and the development of suitable preventative strategies.

A number of caveats and methodological considerations arise from our review of preclinical G × E models relevant to schizophrenia. Firstly, the heuristic value of a G × E model depends upon the level of construct validity possessed by the experimental model of the environmental stressor. Translation of epidemiologically appropriate environmental factors into current animal models of G × E interactions constitutes a particular challenge for models of G × E interplay in schizophrenia [196]. Secondly, it has to be noted that the majority of the studies outlined above have been conducted using rodent models involving a single gene mutation, while schizophrenia is a polygenic disorder [5]. Thirdly, much of the evidence outlined in the preceding sections is essentially descriptive, or the studies cited have focused on a limited number of molecular markers; more detailed molecular interrogation of phenotypic effects, at different time points, is required. In particular, neural circuits in animal models of G × E interactions will need to be examined with respect to behavioural changes, with a particular attention to the pathological trajectory from early development to the emergence and expression of the specified disease-relevant endophenotypes in adulthood [5]. These mechanistic studies will provide a solid basis for the development and evaluation of targeted preventative or rescue strategies. Lastly, several of the G × E models discussed have demonstrated that the effects of coexposure to a genetic mutation and an environmental stressor can result in modification of the phenotypic effects of one factor or the other but may also produce phenotypic effects, both protective and adverse, which may not be observed following exposure to any one factor alone [28].

It has been suggested that genetic risk to develop a psychotic disorder may be expressed as altered responsivity to everyday stressful situations [197], such that idiopathic responsivity to stressors may be an important determinant of induction of psychosis. At a phenotypic level, both the human genetic and preclinical G × E data related to schizophrenia have highlighted the importance of incorporating behavioural and physiological measures of stress responsivity in any phenotyping strategy. Both streams of evidence have clearly shown that it represents a modulating trait which might increase risk for schizophrenia [198, 199] and modulate the expression or severity of schizophrenia-relevant endophenotypes in preclinical G × E models (e.g., [24]).

As evident in the above description of G × E interaction in relevant mutant models, sex-specific effects are commonly observed, even allowing for the limited number of studies which have examined such effects in both sexes. Gender differences in schizophrenia have been noted across such domains as symptomatology and course of illness. Males show lower premorbid functioning, earlier age of onset, more severe cognitive deficits, and poorer prognosis at an earlier age of onset, and a poorer course of illness [200, 201]. There is sufficient evidence to conclude that independent and interactive effects of genetic and environmental manipulations on behavioural indices can differ between the sexes. Therefore, there is a requirement for G × E models to be validated for both sexes.

Despite the difficulty in interpreting the evidence for first- and second-order interactions arising from multifactorial G × E studies conducted in nonhumans, some authors have proposed common biological mechanisms or processes which might underlie such interactions [5]. One such mechanism is a disturbance in glutamatergic function, which may be related to dysfunction of parvalbumin-positive interneurons in the cerebral cortex and hippocampus, which are sensitive to alterations in NMDA-type glutamate receptors [202]. One of the common findings in both animal models and postmortem tissue from patients with schizophrenia is a reduction of mRNA or protein levels of the calcium-binding protein parvalbumin in cortical fast-spiking (FS) interneurons. Both preclinical genetic and environmentally based models using schizophrenia risk genes or stressors, respectively, have consistently observed a decreased number or impaired function of parvalbumin-positive interneurons in the hippocampus or cortex [91, 203]. A different model has suggested that genetic risk factors interact with social environmental risk factors (including early life adversity and psychosocial stress) to impact on the DA system, increasing its response to environmental stressors and to the abuse of drugs such as cannabis and psychostimulants [204, 205]. There are various strands of evidence to support this theory, including the well-characterised impact of acute and long-term exposure to stress and drugs of abuse on mesolimbic DA-ergic pathway dysfunction, and the fact that many of the genetic risk factors implicated in schizophrenia are associated with underlying alterations in the DA system [206]. Mesolimbic DA-ergic dysregulation is posited to be a fluid and dynamic process that may be reactive to acute and chronic stressors, including early brain insult, prolonged exposure to drugs of abuse, and psychosocial stress, across the lifespan of the individual. Another theory places a greater emphasis on the convergence of genetic and environmental factors upon regulation of synaptic plasticity and function, as well as the stabilisation of cortical microcircuitry [42, 207]. It has been observed that intact synaptic function depends on a large number of molecular pathways which will be affected by several environmental factors throughout brain development. Additionally, stress-associated signalling cascades are well known to modulate the development and maintenance of synaptic connectivity [5].

What existing animal studies of G × E interactions relevant to schizophrenia highlight is that developing valid multifactorial models which are amenable to investigations not yet possible in clinical studies will become increasingly important in determining the mechanisms underlying convergence of genetic and environmental risk factors and their interaction.

## Figures and Tables

**Table 1:** Summary of evidence for gene, environment, and gene × environment effects in mutant models for selected genes associated with schizophrenia.

Gene target	Environmental exposure	Reference(s)	Impact on schizophrenia-relevant behavioural endophenotypes			Use of preventative or rescue strategy
			Genetic manipulation	Environmental manipulation	Gene × environment	
*NRG1*	Prenatal Poly I:C	[92]	Decreased social novelty preference and PPI; sex-specific (females only) decrease in working memory	Disruption of working memory and PPI	Decreased sociability in Poly I:C × WT mice only; sex-specific decrease in alternation (i.e., working memory) following Poly I:C treatment was attenuated in female *NRG1* mutants	—

*NRG1*	Acute Δ-9 THC during adulthood	[128, 129]	Increased novelty-induced activity; decreased anxiety in the elevated plus maze and light-dark test; increased *c-fos* expression in the lateral septum and nucleus accumbens	Decreased novelty-induced activity; increased anxiety in the elevated plus maze; enhanced PPI; decreased social interaction; increased *c-fos* expression in the dorsolateral part of the bed nucleus of the stria terminalis and central nucleus of the amygdala	Increased sensitivity to locomotor suppressant effects of THC in *NRG1* mutants; greater PPI enhancement in *NRG1* mutants; greater increase in *c-fos* expression in the dorsolateral part of the bed nucleus of the stria terminalis and central nucleus of the amygdala, and paraventricular nucleus of the hypothalamus in *NRG1* mutants	—

*NRG1*	Subchronic CP 55, 940 [CB1R agonist] during adulthood	[131]	Increased novelty-induced activity	Decreased novelty-induced activity; increased anxiety in elevated plus maze and open field	Increased tolerance to CP55,940-induced anxiolytic and locomotor suppressant effects in *NRG1* mutants; increased *c-fos* expression in lateral septum in treated *NRG1* mutants	—

*NRG1*	Subchronic Δ-9-THC during adolescence	[132]	Increased novelty-induced activity	Decreased novelty-induced activity	Decreased anxiogenic effects of THC in *NRG1* mutants; decreased social investigative behaviours in WT only; disruption of PPI in THC-treated *NRG1* mutants	—

*NRG1*	Subchronic cannabidiol during adulthood	[136]	Increased novelty-induced activity; disrupted PPI; decreased 5-HT_2a_ receptor binding in substantia nigra	Enhanced PPI after acute cannabidiol; increased social interaction following chronic cannabidiol	Decreased sensitivity to anxiolytic effects of cannabidiol in mutants; selective enhancement of social interaction and PPI in *NRG1* mutants; selective enhancement of GABA_A_ receptor binding in the granular retrosplenial cortex of *NRG1* mutants and reduction of 5-HT_2a_ receptor binding in the substantia nigra of WT	—

*NRG1*	Subchronic Δ-9 THC during adolescence	[133]	Altered expression of proteins involved in vesicular release of neurotransmitters, 5-HT neurotransmission, and growth factor expression	Reduced hippocampal expression of heat shock proteins and oxidative stress	Altered expression of proteins implicated in NMDA-mediated glutamatergic neurotransmission	—

*NRG1*	Social defeat during adolescence	[86]	Increased novelty-induced activity; decreased social novelty preference; PPI disruption; decreased anxiety	—	Selective decrease in anxiety and working memory in stressed *NRG1* mutants; protective effect of *NRG1* genotype on disruption of sucrose preference following social defeat	—

*NRG1*	Chronic restraint stress during adolescence	[153]	—	Increased NMDA receptor binding in ventral part of the lateral septum and dentate gyrus	PPI disruption in *NRG1* only following chronic stress exposure; altered patterns of NMDA receptor binding in infralimbic subregion of medial prefrontal cortex and dentate gyrus; decreased corticosterone levels, as well as increased apical dendritic spine density and decreased apical dendritic lengths and complexity in layer II/III pyramidal neurons of the medial prefrontal cortex	—

*DISC1*	Prenatal Poly I:C	[50]	Enlargement of the lateral ventricles	Increased anxiety in open field; decreased volume of amygdala and left/right periaqueductal grey; decrease in linear density of spines in pyramidal neurons of the CA1 region	Increase in anxiety in elevated plus maze and increased immobility in forced swim test in *DISC1* mutants; decreased social interaction in challenged *DISC1* offspring; decreased linear spine density on dendrites of granule cells of the dentate gyrus in *DISC1* mutants only; opposite effects on lateral ventricle volume (increased in WT, decreased in mutants)	—

*DISC1*	Prenatal Poly I:C	[104]	Decreased PPI in *DISC1* Q31L mutant; decreased LI and social affiliative behaviour in *DISC1* L100P line	Decreased PPI and LI; disruption of spatial discrimination and object exploration	More prominent PPI and LI deficits in L100P mutants; impaired working memory and sociability in challenged *DISC1* offspring; increase of Poly I:C-induced increase in IL-6 in brains of *DISC1* mutants	Coadministration of IL-6 antagonist with Poly I:C reversed Poly I:C-related deficits in mutants and controls

*DISC1*	Neonatal Poly I:C	[105, 106]	—	—	Selective deficits in short-term memory and object recognition memory in *DISC1* mutants; increased behavioural sensitivity to MK-801 in *DISC1* mice exposed to Poly I:C; selective decrease in parvalbumin-positive interneurons in the medial prefrontal cortex	Cognitive deficits in Poly I:C-treated DISC1 mutants improved by clozapine while haloperidol had no effect; clozapine suppressed the augmentation of MK-801-induced hyperactivity

*DISC1*	Prenatal lead exposure	[107]	Enlargement of lateral ventricles; decreased anxiety in open field	Increased anxiety in open field; increased anxiety in elevated plus maze; increased MX-801 responsivity; decreased PPI; enlargement of lateral ventricles	Heightened responsivity to the NMDAR antagonist MK-801 and increased PPI disruption in female *DISC1* mice; synergistic decrease in exploratory activity and synergistic increase in lateral ventricular volume in *DISC1* mutants	Systemic administration of D-serine, a coagonist at the NMDA receptor, reversed PPI deficits in female lead-exposed mutants

*DISC1*	Subchronic Δ-9 THC during adolescence	[137]	Decrease in contextual fear memory; decreased synaptic CB1R expression in the prefrontal cortex, hippocampus, and amygdala	Decrease in synaptic CB1R expression in the prefrontal cortex, hippocampus, and amygdala	Disruption in cue-dependent fear memory	—

*DISC1*	Social defeat during adulthood	[154]	Decreased PPI in *DISC1 *L100P; impaired LI in L100P and *DISC1* Q31L; decreased sociability and social novelty in Q31L mutants	Increased immobility in forced swim test; decreased sucrose intake in the sucrose consumption test	Decrease in exploratory activity and sociability and social novelty in L100P; increase in anxiety in the elevated plus maze in L100P but not Q31L mutants exposed to social defeat	—

*DISC1*	Prolonged social isolation during adolescence	[51, 155]	—	—	PPI disruption, forced swim immobility, and methamphetamine-induced locomotion, in isolated *DISC1 *mutants; decreased tyrosine hydroxylase expression, total tissue DA levels, and DA in the frontal cortex; increased DA release in the nucleus accumbens; altered DNA methylation of *tyrosine hydroxylas*e, *BDNF*, and *FK506 binding protein 5* genes	RU-486 normalized basal and methamphetamine-induced extracellular DA, tyrosine hydroxylase, and DA D2 receptor levels in G × E model; RU-486 also reversed PPI, forced swim test deficits, and changes in amphetamine-induced activity in this model

*COMT*	Subchronic Δ-9 THC during adolescence	[123]	Improved spatial working memory in *COMT* KO males	Decreased object recognition, social novelty preference, and anxiety	Increased hyperactivity and greater disruption of working memory in THC-treated *COMT* KO mice	—

*COMT*	Subchronic Win 55,212 [CB1R agonist] during adolescence	[143]	—	Decreased social novelty preference; decreased anxiety in the light-dark test	Selective disruption of PPI in cannabinoid-treated *COMT* mutants; decreased sensitivity to disruptive effects on sociability in mutants relative to WT	—

*COMT*	Subchronic Δ-9 THC during adolescence	[144]	Increased CB1R intensity in the prefrontal cortex; decreased CB1R intensity in the hippocampus; parvalbumin cell size decreased in *COMT* heterozygotes	Decreased cell density in the VTA	Decreased parvalbumin cell intensity in the prefrontal cortex; decreased DA cell size in VTA; increased CB1R intensity in hippocampus of THC-treated *COMT *mutants	—

BDNF, brain-derived neurotrophic factor; CB1R, cannabinoid receptor 1; *COMT*, catechol-*O*-methyltransferase; DA, dopamine; Δ-9 THC, delta-9-tetrahydrocannabinol; *DISC1*, disrupted in schizophrenia 1; GABA_A_, gamma-aminobutyric acid type A receptor; IL-6, interleukin 6; KO, knockout; LI, latent inhibition; NMDA receptor, N-methyl-D-aspartate receptor; NRG1, neuregulin-1; PPI, prepulse inhibition; 5-HT_2A_, serotonin 2_A_ receptor; VTA, ventral tegmental area.

**Table 2:** Summary of evidence for gene, environment, and gene × environment effects in mutant models for selected genes associated with schizophrenia.

Gene target	Environmental exposure	Reference(s)	Impact on schizophrenia-relevant behavioural endophenotypes			Use of preventative or rescue strategy
			Genetic manipulation	Environmental manipulation	Gene × environment	
*Nurr1*	Prenatal Poly I:C	[110]	Increased novelty-induced activity; decreased PPI, reduction in tyrosine hydroxylase-positive cells in the substantia nigra	Increased novelty-induced activity; decreased PPI; spatial working memory deficits; increase in tyrosine hydroxylase-positive cells in the VTA	Additive effects on novelty-induced hyperactivity; synergistic reduction in attentional shifting and sustained attention; decrease in DA D2 receptor immunoreactivity in the nucleus accumbens	—

*Snap-25*	Variable prenatal stress	[183]	Decreased PPI in the *blind-drunk* point mutant	PPI disruption	Decreased social novelty preference	Clozapine and haloperidol (to a lesser extent) reversal of PPI deficits was most pronounced in G × E group

*Snap-25*	Prenatal nicotine exposure	[184]	Increased novelty-induced activity and decreased social interaction	—	More pronounced novelty-induced hyperactivity and greater disruption of social interaction; deficits in DA D2 receptor-dependent induction of long-term synaptic depression	—

*BDNF*	Chronic methamphetamine exposure	[188]	—	Locomotor sensitisation and increased entropy	Decreased locomotor sensitisation and entropy in *BDNF* heterozygotes	—

*BDNF*	Chronic methamphetamine exposure	[189]	Decreased PPI and increased acoustic startle reactivity in *BDNF* heterozygotes	Locomotor sensitisation; increased sensitivity to MK-801 and amphetamine-induced PPI disruption	Increased sensitivity to amphetamine-induced PPI disruption in preexposed *BDNF* heterozygotes	—

*RELN*	Maternal separation	[193]	Decreased frequency of ultrasonic vocalisations; decreased activity in a novel environment	—	Decreased sensitivity to disruptive effects of maternal separation in heterozygous *RELN *mutants	—

*RELN*	Prenatal exposure to the pesticide chlorpyrifos Maternal separation	[192]	Decreased frequency of ultrasonic vocalisations	—	Prenatal chlorpyrifos: selective increase in ultrasonic vocalisation in *RELN *mutants; disrupted behavioural response to acute scopolamine Maternal separation: decreased social motivation in WT but not *RELN* mutants	—

*RELN*	Prenatal hypoxia	[194]	Increase in frontal cortex volume in *RELN* mutants	Reduction in glucocorticoid receptor protein levels in frontal cortex	Increase in frontal cortex volume in WT but opposite effect observed in *RELN* mutants; selective reduction in glucocorticoid receptor protein levels in hippocampus of *RELN* mutants; selective changes in brain expression of hypoxia-related proteins in mutants	—

*BDNF*, brain-derived neurotrophic factor; DA, dopamine; Δ-9 THC, delta-9-tetrahydrocannabinol; NURR1, nuclear receptor related 1 protein; PPI, prepulse inhibition; *RELN*, reelin; SNAP-25, synaptosome associated protein 25 kDa; VTA, ventral tegmental area; WT, wildtype.
